# Case of Spontaneous Ante-partum Hæmorrhage—Slight Symptoms Proportionate to Amount of Blood Lost

**Published:** 1881-04

**Authors:** G. Frank Lydston

**Affiliations:** House Surgeon Charity Hospital, N. Y.


### Article IV.

Case of Spontaneous Ante-partum Hæmorrhage—Slight Symptoms Proportionate to Amount of Blood Lost. By G. Frank Lydston, m. d., House Surgeon Charity Hospital, N. Y.

The patient, K. S., is a primipara, and had always enjoyed excellent health previous to pregnancy. Is a robust woman, twenty-four years of age, and apparently in the best of health. There is no history of venereal disease, or intemperence, but patient states that she has had leucorrhœa to a slight degree at times. The date of last menstruation, was February 22, 1880. Patient began to have gastric disturbances about the middle of March, and this has persisted up to time of labor, causing considerable annoyance, but with no apparent deleterious effect upon her general health. States that she has passed less urine than formerly. No other trouble was manifested until Oct. 1st. On this date, at about 10 a. m., patient had a slight attack of syncope, consciousness not being completely lost, but patient being compelled to lie down. She stated that she had caught a severe cold, and felt faint and dizzy. She appeared to have a slight coryza, and the surface was cold, being covered with a cold perspiration. None of thesymptoms, however, were especially marked. No pain whatever was complained of. Patient was ordered to bed, and gr. x of Dover's powder administered, and, on the following morning she arose feeling quite well. On Oct. 3d, at 3 p. m., being the third day after trouble above mentioned, patient began having slight pains, but said nothing about them, until 6 p. m., at which time I was called and found os fully dilated. No presentation could be felt. At 6.55 membranes were ruptured and child, descended in pelvis, the breech presenting in R. D. A. position. The lower limbs and breech were delivered at 8 p. m., and there being some delay in delivery of head, it was extracted. The placenta was retained until 9 p. m. when it was removed by hand. The child was dead, and the epidermis had become macerated so that it came off readily in large patches, showing that death had occurred several days before labor. At the time the breech engaged in the pelvic brim, a small soft mass was felt protruding by its side, and posteriorly. This descended, and was expelled simultaneously with the head, proving to be a firm clot, evidently several days old, and sufficiently organized to retain its integrity under a quite forcible stream of water. It was found to weigh 23½ oz. av. No definite cause for this hæmorrhage could be discovered, although the placenta appeared to be slightly waxy, not being however examined microscopically. An autopsy was held upon the child, but nothing was discoverable, save a small amount of amyloid deposit in the liver.

The patient vomited a greenish fluid shortly after delivery, but said that she felt better as a consequence. She manifested no untoward symptoms subsequently, and was convalescent on 10th day.

The case appears rather peculiar and somewhat interesting, in view of some of the facts above mentioned. Here was a woman in perfect health apparently, who suffered from a profuse accidental hæmorrhage, coming on with no evident exciting cause. She was perfectly quiet at the time, if, as I infer, the slight attack of syncope which she had several days before labor, was the result of loss of blood. Although of a full habit, it is singular that the patient could lose such an amount of blood and present such slight symptoms. There may have been syphilis in the case ; the slight waxy changes in placenta, and in the liver of the child, may have been the results of that disease, still the mother had neither history nor evidences of it. This would not exclude syphilis, however, as the father may have been at fault, as is now pretty generally held in regard to hereditary syphilis. This waxy change existing, it is possible that the retching and vomiting which occurred on the morning of the attack of syncope, caused a partial placental detachment, with hæmorrhage as consequence. It is remarkable that so large a clot, acting as a foreign body, should not bring on labor immediately, rather than in several days thereafter.

